# Particle-Cell Contact Enhances Antibacterial Activity of Silver Nanoparticles

**DOI:** 10.1371/journal.pone.0064060

**Published:** 2013-05-30

**Authors:** Olesja Bondarenko, Angela Ivask, Aleksandr Käkinen, Imbi Kurvet, Anne Kahru

**Affiliations:** 1 Laboratory of Environmental Toxicology, National Institute of Chemical Physics and Biophysics, Tallinn, Estonia; 2 Department of Chemical and Materials Technology, Tallinn University of Technology, Tallinn, Estonia; Harbin Institute of Technology, China

## Abstract

**Background:**

It is generally accepted that antibacterial properties of Ag nanoparticles (AgNPs) are dictated by their dissolved fraction. However, dissolution-based concept alone does not fully explain the toxic potency of nanoparticulate silver compared to silver ions.

**Methodology/Principal Findings:**

Herein, we demonstrated that the direct contact between bacterial cell and AgNPs' surface enhanced the toxicity of nanosilver. More specifically, cell-NP contact increased the cellular uptake of particle-associated Ag ions – the single and ultimate cause of toxicity. To prove that, we evaluated the toxicity of three different AgNPs (uncoated, PVP-coated and protein-coated) to six bacterial strains: Gram-negative *Escherichia coli*, *Pseudomonas fluorescens*, *P. putida* and *P. aeruginosa* and Gram-positive *Bacillus subtilis* and *Staphylococcus aureus*. While the toxicity of AgNO_3_ to these bacteria varied only slightly (the 4-h EC_50_ ranged from 0.3 to 1.2 mg Ag/l), the 4-h EC_50_ values of protein-coated AgNPs for various bacterial strains differed remarkably, from 0.35 to 46 mg Ag/l. By systematically comparing the intracellular and extracellular free Ag^+^ liberated from AgNPs, we demonstrated that not only extracellular dissolution in the bacterial test environment but also additional dissolution taking place at the particle-cell interface played an essential role in antibacterial action of AgNPs. The role of the NP-cell contact in dictating the antibacterial activity of Ag-NPs was additionally proven by the following observations: (i) separation of bacterial cells from AgNPs by particle-impermeable membrane (cut-off 20 kDa, ∼4 nm) significantly reduced the toxicity of AgNPs and (ii) *P. aeruginosa* cells which tended to attach onto AgNPs, exhibited the highest sensitivity to all forms of nanoparticulate Ag.

**Conclusions/Significance:**

Our findings provide new insights into the mode of antibacterial action of nanosilver and explain some discrepancies in this field, showing that “Ag-ion” and “particle-specific” mechanisms are not controversial but, rather, are two faces of the same coin.

## Introduction

Ag nanoparticles (AgNPs) are the first commercialized NPs that are nowadays used as broad-spectrum antimicrobials in over 300 consumer products including cosmetics, clothing, detergents, dietary supplements, water filters, electronics and children's toys [1], [2]. Actually, colloidal silver, e.g., protein-stabilized nanosized Ag particles, has been used for numerous medical purposes already since the late 19th century [3], [4]. To date, approximately 500 tons of nanosilver is produced annually and there is a high risk for environmental pollution [5] due to its leaching from the nanosilver-containing consumer products as well as through industrial waste streams, mainly *via* waste and sewage treatment plants [6], [7], [8]. Currently, most of the studies on nanosilver toxicity to bacteria focus on a laboratory model bacterium *Escherichia coli* and on human pathogens such as *Staphylococcus aureus* and *Pseudomonas aeruginosa*. Remarkably less information is available for environmentally relevant bacterial species such as *Pseudomonas putida* and *P. fluorescens* (Figure S1).

Despite of numerous publications, the antimicrobial mechanism of AgNPs is still under debate. It is generally acknowledged that the size and the specific surface area affect the antibacterial activity of AgNPs [9], [10]. More specifically, recent publications have revealed that the toxicity of AgNPs to *E. coli* is proportional to the relative surface area of silver oxide monolayers, which dissolve and release Ag ions upon contact with water [11]. Accordingly, AgNPs lacking oxidized surfaces (and thus, not dissolving) proved not toxic to bacterial cells, suggesting that the toxicity of AgNPs is ultimately dictated by released Ag ions [12], [13].

In contrast, numerous studies have found that Ag^+^ concentrations released from AgNPs into the soluble phase during toxicity assays were too low to explain the observed antibacterial effects [14], [15], [16], [17], [18], [19]. As a rule, in these assays the dissolution of AgNPs was quantified by atomic absorption spectroscopy (AAS) or inductively coupled plasma spectrometry (ICP) after the separation of nanoparticulate Ag by ultracentrifugation or ultrafiltration. The toxicity of residual soluble Ag in the supernatant/filtrate was further compared with the toxicity of Ag ion applied as a soluble salt. Such an experimental setup assumes that Ag ions are equally distributed outside of bacteria (i.e., in the test medium) and inside the bacterial cells and that the bioavailability of Ag ions to bacterial cells from the surface of AgNPs and from the aqueous phase is similar.

Hereby we hypothesized that AgNPs, even if not toxic *per se*, may serve as efficient carriers of toxic Ag ions. Using a suite of six Gram-positive and Gram-negative bacterial strains, three types on AgNPs with different coatings and three different techniques to determine the dissolution of these AgNPs, we demonstrated the significance of the direct contact between bacterium and NPs' surface. We showed for the first time and quantitatively that the intracellular bioavailable fraction and hence, toxicity of Ag ions from AgNPs could not be always predicted from the conventional studies that measure extracellular dissolution. Rather, the intracellular concentration of silver ions inside the bacterial cells – the ultimate determinant of AgNPs toxicity – was dictated by both, extracellular dissolution of AgNPs and additional dissolution at bio-nano interface. Thus, our results aim to resolve the main controversy behind the Ag ion-related toxicity mechanism of silver nanoparticles.

## Materials and Methods

### Chemicals and Nanoparticles

All the purchased chemicals were at least of analytical grade. AgNO_3_ was from J.T.Baker, uncoated AgNPs (nAg, primary size 30–100 nm) were from Sigma-Aldrich (CAS number 7440-22-4), protein (casein)-coated colloidal AgNPs (nAg-Col, primary size 5–30 nm; [20]) were from Laboratorios Argenol S. L. (batch N° 297). The manufacturer-provided characteristics of nAg-Col were verified: (i) molecular weight of the casein coating was confirmed using UPLC size exclusion chromatography, which identified homogeneous a 5 kDa protein and (ii) the concentration of casein coating was verified using Pierce BCA Protein Assay Kit (Thermo Scientific) (data not shown). Polyvinylpyrrolidone-coated AgNPs (nAg-PVP, primary size 8–11 nm) were synthesized and characterized as described in [21].

The stock solutions of all Ag formulations (1 000 mg Ag/l, 20 ml) were prepared in distilled (DI) water (pH = 5.8) and further stored in the dark at +4°C. The stock solutions of nAg and nAg-PVP were homogenized using ultrasonic probe (40 W, 3 minutes; 450 Ultrasonifier, Branson Ultrasonics Corporation, USA) once after preparation.

The primary particles of AgNPs were visualized by transmission electron microscopy (TEM, SUMY-SELMI, EM-125) and the particle size was measured using ImageJ software. Size distribution of AgNPs (Figure S2) was calculated based on 65 particles. Hydrodynamic size (at ζ-average) and ζ-potential of AgNPs were measured at a concentration of 100 mg/l immediately after AgNPs' dispersion in DI water and in the bacterial growth medium using Malvern Zetasizer (Nano-ZS, Malvern Instruments, UK).

### Quantification of Dissolved Silver

The dissolution of AgNPs in DI water as well in the bacterial growth medium was measured using three different techniques that enable to determine the extracellular free Ag^+^, extracellular dissolved Ag and intracellular Ag^+^. **Extracellular free Ag^+^** was measured from the suspensions of AgNPs with Ag-ion selective electrode (Ag-ISE) (Van London-pHoenix Company). **Extracellular dissolved Ag** was determined from the supernatants that were obtained after ultracentrifugation of AgNPs' suspensions at 390 000 g for 60 minutes. According to the calculations, under these conditions all AgNPs and Ag-protein complexes with the molecular mass above 5 kDa should settle [22]. The supernatants were analyzed by atomic absorption spectroscopy (AAS) in a certified laboratory of Tallinn University of Technology, Estonia, applying the standard EVS-EN ISO/IEC 17025∶2005. **Intracellular Ag^+^** from AgNPs was quantified with recombinant bioluminescent *Escherichia coli* MC1061 (pSLcueR/pDNPcopAlux) Ag-biosensor [23] as described below.

### Bacterial Growth Inhibition Assay

For the growth inhibition assay bacteria (Gram-negative *Escherichia coli* MC1061, *Pseudomonas fluorescens* OS8, *P. putida* KT2440, *P. aeruginosa* DS10-129 and Gram-positive *Bacillus subtilis* BR151 and *Staphylococcus aureus* RN2440; Table S1) were cultivated overnight on a shaker (200 rpm, 30°C) in 3 ml of modified (NaCl-free) LB medium (10 g tryptone and 5 g yeast extract *per* liter, pH = 7). NaCl was not added to the medium to avoid the formation of insoluble AgCl. Before the test, bacterial culture was diluted in NaCl-free LB medium to OD_600_ = 0.05 corresponding to approximately 10^6^ cells/ml. Bacterial growth inhibition assay was conducted on transparent sterile 96-well Cliniplates (Thermo Labsystems). Briefly, 100 µl of the dilution of AgNPs or AgNO_3_ (from 0.01 to 100 mg Ag/l) in DI water (sample) or pure DI water (control) was pipetted onto the wells. Then, 100 µl of bacterial culture in NaCl-free LB medium was added. The test plates were incubated on a plate shaker (Heidolph Titramax 1000, 350 rpm) at 30°C for 4 hours. Absorbance of the bacterial cultures at 600 nm (OD_600_) was measured in 1 h intervals with Multiskan plate reader (Thermo Scientific). The bacterial growth inhibition (INH%) was calculated as follows:
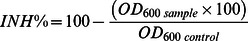
In the end of the growth inhibition assay, 3 µl of each sample was removed and plated onto LB agar plates to assess the viability of the cells. The plates were incubated at 30°C for 24 h and the minimum bactericidal concentration (MBC) was characterized as the lowest concentration of Ag compound where no colonies were observed, i.e., the concentration that resulted in irreversible inhibition of the bacterial growth.

To study the role of the direct particle-cell contact in the antibacterial effects of AgNPs, additional experiments where bacteria were separated from AgNPs by 20 kDa (∼4 nm, [24]) dialysis membrane (Slide-A-Lyzer MINI Dialysis Device, 20K MWCO, Thermo Scientific) were performed. In this case, 400 µl of bacterial suspension was pipetted onto the wells of 48-well transparent cell culture plates (nontreated polystyrene, BD Falcon). Then, polypropylene cups with the dialysis membrane on the bottom were inserted into the wells and 400 µl of AgNPs/AgNO_3_ (sample) or DI water (control) were pipetted into the cups. Bacteria were grown at 30°C, 750 rpm for 4 hours and OD_600_ was measured.

### Quantification of Intracellular Silver Ions

Quantification of intracellular Ag ions was performed using recombinant biosensor bacteria *Escherichia coli* MC1061 (pSLcueR/pDNPcopAlux). The response of this recombinant *E. coli* to intracellular Ag ions is mediated *via* CueR activator protein and its regulated *copA* promoter that is fused to the bioluminescence encoding genes. Therefore, in the sub-toxic region, the presence of intracellular Ag ions leads to the increase of bioluminescence of these recombinant bacteria in a dose-dependent manner [23].

The preparation of test bacteria and the procedure of the biosensor assay was analogous to the bacterial growth inhibition assay with the following exceptions: (i) the growth medium of bioluminescent Ag-biosensor *E. coli* MC1061 (pSLcueR/pDNPcopAlux) was supplemented with 100 µg/l ampicillin and 10 µg/l tetracycline during overnight cultivation to maintain the recombinant plasmids and (ii) the assay was conducted on white 96-well Cliniplates with transparent bottom that allowed the determination of both, luminescence and optical density in parallel. Multiskan plate reader (Thermo Scientific) was used for optical density and Orion II plate luminometer (Berthold Detection Systems) for the bioluminescence measurement. Briefly, 100 µl of bacterial suspension was exposed to 100 µl of 0.01–100 mg Ag/l dilutions of AgNO_3_ or AgNPs in DI water (sample) or DI water (background) at 30°C for 4 hours. Dose-response curves of the Ag-biosensor were obtained by plotting the applied concentrations of Ag against the bioluminescence of Ag-biosensor (as fold induction) in respective samples. Fold induction was calculated as follows:
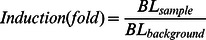
where BL_sample_ is the bioluminescence of Ag-biosensor in the sample and BL_background_ is the background bioluminescence. Intracellular Ag was determined by using the log-log linear regression equations derived from the linear region of the dose-response curves of Ag-biosensor to AgNO_3_ and AgNPs , whereas AgNO_3_ was considered 100% bioavailable and was used as a standard (Figure S3).

### Assessment of Cell-Nanoparticles Interaction

Bacterial suspensions were cultivated overnight on a shaker (200 rpm, 30°C) in 3 ml of NaCl-free LB medium. Then, the bacterial cultures were diluted in NaCl-free LB medium to OD_600_ = 0.4. 700 µl of diluted bacterial suspension was added to 700 µl of 20 mg Ag/l nAg-Col in DI water (final concentration of nAg-Col in the test 10 mg Ag/l) and immediately centrifuged at 4 000 g for 5 minutes. 1 ml of obtained supernatants was used to measure the UV-visible (UV-Vis) wavelength absorption spectra with a Thermo Multiskan Spectrum (Thermo Electron Corporation, Finland). As a control, the spectrum of 10 mg/l collargol in half-strength NaCl-free LB medium centrifuged at 4 000 g for 5 minutes was analyzed.

### Statistical Analysis

All experiments were performed in at least three biological replicates and the data were expressed as mean ± standard deviation. To define statistically significant differences, the data were analyzed either with one way analysis of variance ANOVA or with unpaired two-tailed *t*-test assuming equal variances at p<0.01.

## Results

### Characteristics of Silver Nanoparticles

**Figure 1:**
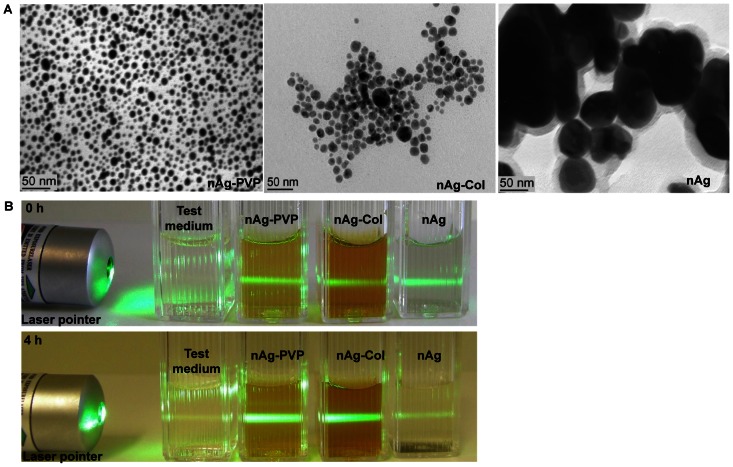
Characterization of silver nanoparticles. A: Transmission electron microscopy images of AgNPs. TEM image of collargol is reprinted from [21] with the permission of Springer. B: Stability of 10 mg/l nanoparticles suspensions in test medium (half-strength NaCl-free LB medium). After 0-h incubation (upper panel), all AgNPs suspensions scattered light upon the illumination by the laser pointer, indicating the presence of particles. After 4-h incubation (lower panel), the light scattering from uncoated nAg was negligible (comparable to that of the test medium with no AgNPs added) and settling of the particles was observed.

The main rationale behind the selection of Ag nanoparticles for this study was their different surface modification. While the Sigma-Aldrich Ag nanoparticles (nAg) had no coating, collargol (nAg-Col) particles were coated with protein (casein) and the nAg-PVP AgNPs had polyvinylpyrrolidone coating (Table 1). All the used AgNPs were spherical (Figure 1A) and had negative ζ-potential (Table 1). Collargol formed the most stable dispersion in the test medium used for bacterial growth inhibition assays (half-strength NaCl-free LB). The average hydrodynamic diameter, D_h_, of nAg-Col particles was 53 nm and the preparation was relatively monodisperse (pdi 0.2). The efficient dispersion of nAg-Col was most likely due to the steric hindrance and electrostatic repulsion between the casein molecules coating these Ag particles. Compared to nAg-Col, the hydrodynamic size of nAg-PVP was larger (D_h_ in the test media = 139 nm, pdi 0.2). This was most probably due to the thick PVP coating and almost neutral surface charge of these particles (Table 1). Both coated AgNPs did not aggregate in the test medium during the test, whereas uncoated nAg formed large aggregates (D_h_ = 269 nm, pdi = 0.7), which settled and formed visible macroscopic silver after 4-h incubation (Figure 1B).

**Table 1:** Characterization of silver nanoparticles (AgNPs) used in the current study.

	nAg	nAg-Col	nAg-PVP
**Coating**	uncoated	casein (30%)^1^	polyvinylpyrrolidone (71%)^1^
**Average primary size** 2 **, nm**	85.7±29.3	14.6±4.7	10.5±4.3
**D_h_** 3 **in DI water, nm**	89 (pdi^4^ = 0.6)	44 (pdi = 0.2)	122 (pdi = 0.2)
**D_h_ in test medium** 5 **, nm**	269 (pdi = 0.7)	53 (pdi = 0.2)	139 (pdi = 0.2)
**ζ-potential in test medium, mV**	−25.5	−26.1	−4.5
**Dissolution** 6 **in DI water, %**	0.82	7.6	36.5
**Dissolution** 6 **in test medium, %**	0.48	2.6	4.4
**Source/Reference**	Sigma-Aldrich	Laboratorios Argenol	[21]

1 Mass fraction of the coating material, analyzed by thermogravimetry [21];

2 Measured from TEM micrographs using ImageJ software (n = 65);

3 Hydrodynamic size, measured by dynamic light scattering (DLS) immediately after dispersion of silver nanoparticles;

4 Pdi states for polydispersity index;

5 Test medium states for half-strength NaCl-free LB;

6 Analyzed by atomic absorption spectroscopy from supernatants of ultracentrifuged (390 000 g×60 min) 10 mg/l AgNPs' suspensions after 4-h incubation at 30°C.

The dissolution rates of AgNPs in both DI water and in the test medium were in the following order: nAg-PVP>nAg-Col>nAg, showing that nAg that formed large aggregates was the least soluble (Table 1). However, despite of its bigger hydrodynamic size, nAg-PVP dissolved better than nAg-Col, indicating that in addition to size, dissolution of AgNPs was also determined by the type of coating. Interestingly, chemical analysis (AAS) revealed that the dissolution rate of all AgNPs was higher in DI water than in test medium (Table 1). Also, according to AAS analysis no additional dissolution of AgNPs in test medium took place during 4 h (insets in Figure S4). At the same time, when 0-h and 4-h UV-Vis spectra of AgNPs in test medium were compared, a decrease in absorption peak height that reveals particle dissolution was observed (Figure S4). Altogether these results indicated a ligand-enhanced dissolution of AgNPs in test medium, whereas the released Ag ions remained bound to the ligands.

### Different Bacterial Strains Exhibit Similar Dose-Response to AgNO_3_ but not to Silver Nanoparticles

**Figure 2:**
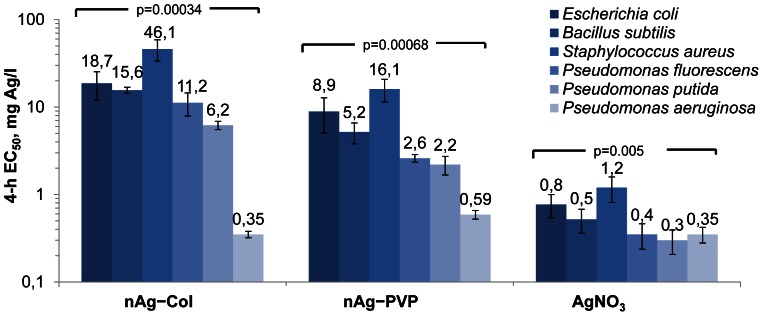
4-h EC_50_ values (endpoint: growth inhibition; note the logarithmic scale) for different silver formulations to bacteria. nAg-PVP states for PVP-coated AgNPs and nAg-Col for collargol. Concentrations are nominal. Data represent the average ± SD (n = 3), p-values denote statistically significant differences between different bacteria (ANOVA).

The growth inhibition curves of the three studied AgNPs and AgNO_3_ to six Gram-positive and Gram-negative bacteria and the respective 4-h EC_50_ values are shown in Figure S5 and Figure 2, respectively. As a rule, both coated AgNPs inhibited bacterial growth at concentrations below 20 mg Ag/l (Figure 2) and at somewhat higher concentrations were bactericidal, i.e., inhibited bacterial growth irreversibly (Table S2). Uncoated nAg, however, had no growth inhibitory effect at tested concentrations except towards *P. aeruginosa* (Figure S5). Altogether, the antibacterial efficiency of AgNPs followed the order nAg-PVP>nAg-Col>nAg, showing a clear positive correlation with their dissolution rates. Notably, the correlation between antibacterial efficiency of AgNPs and their size was less evident, because nAg-PVP NPs with the larger hydrodynamic size were mostly more toxic to bacteria than nAg-Col (Figure 2).

The shapes of the dose-response curves of all bacterial strains to AgNO_3_ (Figure S5) as well as the corresponding 4-h EC_50_ values (Figure 2) were similar revealing similar mechanism of toxicity of ionic Ag. Indeed, the difference between the EC_50_ values of AgNO_3_ to various bacterial strains was only 4-fold: the lowest EC_50_ value was measured for *P. putida* (0.3 mg Ag/l) and the highest for *S. aureus* (1.2 mg Ag/l). However, the shapes of the dose-response curves of various bacterial strains to AgNPs were remarkably different (Figure S5). Especially interesting was the high toxicity of all forms of nanoparticulate Ag to human pathogen *P. aeruginosa*. Even marginally dissolving uncoated Ag inhibited the growth of *P. aeruginosa* (4-h EC_50_ = 11.8 mg Ag/l), but had no effect on other bacterial strains in the concentration range tested (Figure S5). Furthermore, the 4-h EC_50_ value of protein-coated nAg-Col for *P. aeruginosa* was 0.35 mg Ag/l, which was comparable to ionic Ag and should indicate 100% solubility of nAg-Col (Figure 2), although the chemical analysis showed that the dissolution rate of nAg-Col was just 2.6% (Table 1). Notably, the high sensitivity to nAg-Col was characteristic only to *P. aeruginosa*; the 4-h EC_50_ of nAg-Col to e.g., *S. aureus* was 46 mg Ag/l, being as much as 130-fold higher than the value for *P. aeruginosa*.

As the dose-response of various bacteria to AgNO_3_ was similar but different to AgNPs, we proposed that either (i) the toxicity of AgNPs was determined not only by Ag ions, (ii) each bacterial strain had different influence on dissolution of AgNPs or (iii) each bacterial strain modulated differently the uptake of Ag ions dissolved from AgNPs.

### Toxicity of Silver Nanoparticles to Escherichia coli is a Function of Intracellular Ag Ions

First, the effect of Ag ions in toxicity of AgNPs was quantified. For that a recombinant Ag ion-responding biosensor bacterium *E. coli* MC1061 (pSL**cueR**/pDNP**copA**lux) was used. As the bioluminescent response of this bacterium is triggered only by Cu and Ag ions [23], its bioluminescence in our test conditions was proportional to intracellular Ag ions.

**Figure 3:**
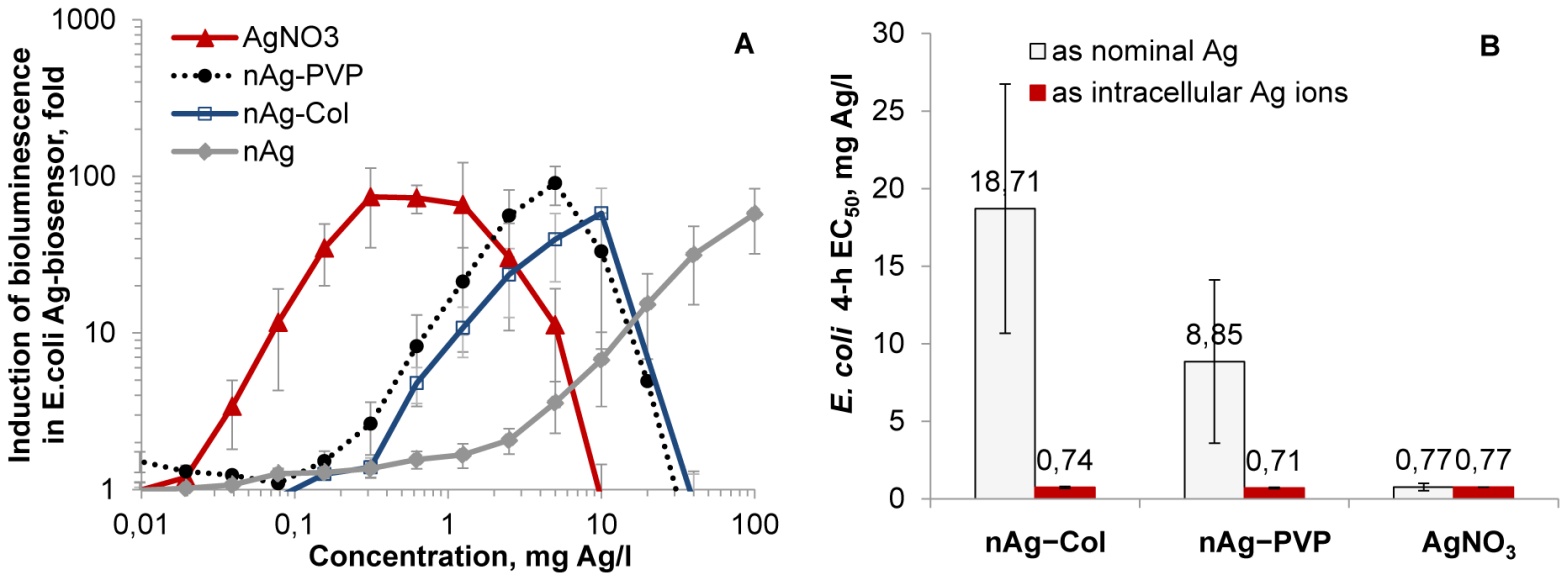
Response of *Escherichia coli* MC1061 to silver formulations. A: Dose-dependent induction of bioluminescence of Ag-biosensor *E. coli* MC1061 (pSLcueR/pDNPcopAlux) in response to silver formulations. The bioluminescence was measured after 4-h exposure (30°C, half-strength NaCl-free LB medium). The decreased bioluminescence at higher concentrations refers to the toxicity. Concentrations are nominal. B: Toxicity (4-h EC_50_) of silver nanoparticles and AgNO_3_ to *E. coli* MC1061. EC_50_ values based on nominal concentrations (open columns) are from Figure 2; EC_50_ values based on intracellular Ag ions (red columns) are re-calculated from Figure 3A. Data represent the average ± SD (n = 3).

The dose-response curves of *E. coli* MC1061 (pSLcueR/pDNPcopAlux) to different Ag formulations are shown in Figure 3A. As expected, the Ag-biosensor was most sensitive to AgNO_3_. Among AgNPs, *E. coli* MC1061 (pSLcueR/pDNPcopAlux) was most sensitive to nAg-PVP, followed by nAg-Col and nAg. The linear region of the sub-toxic part of the dose-response curves (Figure 3A) was used to quantify the intracellular Ag more precisely, revealing that 8.0±1.1% of nAg-PVP, 4.0±0.4% of nAg-Col and 0.6±0.2% of nAg were transformed into intracellular Ag ions. When the nominal *E. coli* 4-h EC_50_ values of Ag formulations (from Figure 2) were re-calculated on the basis of intracellular Ag ions, the resulting 4-h EC_50_ values of both coated AgNPs were very close to that of AgNO_3_: EC_50_ (mg Ag/l) for nAg-Col was 0.74, for nAg-PVP 0.71 and for AgNO_3_ 0.77 (Figure 3B). As the Ag-biosensor *E. coli* MC1061 (pSLcueR/pDNPcopAlux) is induced only by intracellular Ag ions and the toxicity of AgNPs measured using *E. coli* growth inhibition assay integrates all the possible toxic effects of AgNPs (i.e., caused by dissolution, production of reactive oxygen species, lipid peroxidation, membrane damage etc.), it was evident that the toxicity of AgNPs was fully determined by intracellular Ag ions.

### Particle-Cell Contact Enhances Antibacterial Efficiency of Silver Nanoparticles

**Figure 4:**
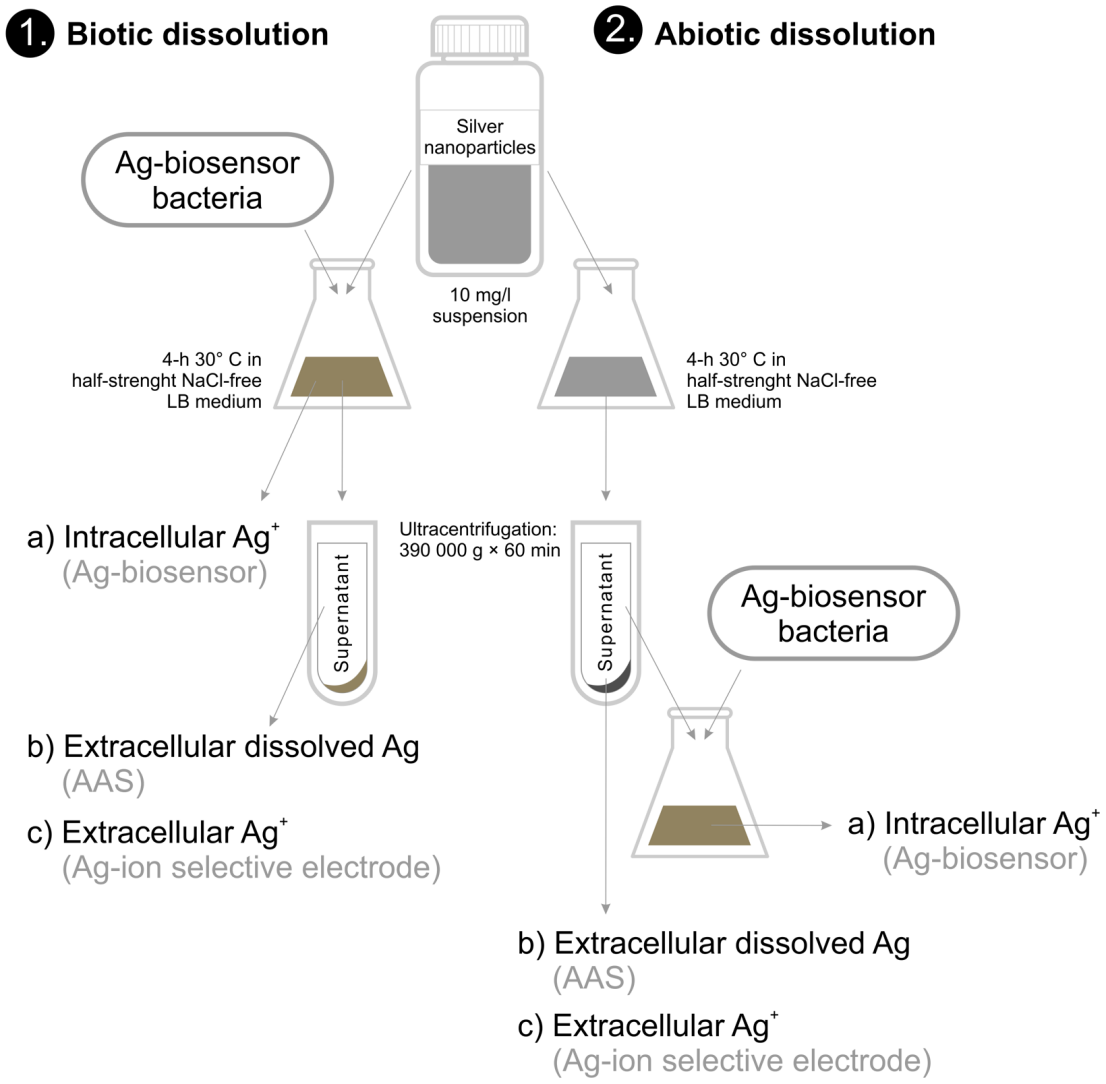
Schematic representation of the experiment to study dissolution of AgNPs. Intracellular Ag ions, extracellular dissolved Ag and extracellular Ag ions were quantified in **biotic** (left) as well **abiotic** (right) conditions. Ag-biosensor *E. coli* MC1061(pSLcueR/pDNPcopAlux) was exposed either to the 10 mg/l suspensions of AgNPs (biotic dissolution) or to the supernatants obtained after ultracentrifugation (390 000 g×60 min) of the respective AgNPs' suspensions (abiotic dissolution). Intracellular Ag ions were quantified as a function of increase of bioluminescence Ag-biosensor *E. coli* MC1061(pSLcueR/pDNPcopAlux), extracellular dissolved Ag was measured by atomic absorption spectroscopy (AAS) and extracellular Ag ions by ion-selective electrode (Ag-ISE).

As the toxicity of AgNPs was mediated *via* intracellular Ag ions (Figure 3) but was orders of magnitude different to different bacterial strains (Figure 2), it was evident that intracellular bioavailability of Ag ions liberated from AgNPs was bacterial strain-specific. We proposed two hypotheses to explain this phenomenon: (i) either each bacterial strain differently modulated the extracellular dissolution of AgNPs *via* bacterial exudates (organic acids, peptides, biosurfactants) or (ii) the cellular uptake of Ag ions *via* cell-NP interaction was different in different bacterial strains. We studied these two hypotheses using *E. coli* cells as an example. For that, we incubated AgNPs with (biotic dissolution) or without (abiotic dissolution) bacterial culture (Figure 4). To test the first hypothesis, we separated the dissolved Ag by ultracentrifugation and compared the biotic and abiotic dissolution rates of AgNPs by quantifying the extracellular dissolved Ag and extracellular free Ag^+^ in the supernatant using AAS and Ag ion-selective electrode, respectively. To test the second hypothesis, we exposed *E. coli* MC1061 (pSLcueR/pDNPcopAlux) Ag-biosensor either to AgNPs' suspensions or to ultracentrifuged supernatants of these suspensions and quantified the internalized Ag ions. Thus, in the former experimental setup *E. coli* cells were in the direct contact with AgNPs and in the latter case, *E. coli* was exposed to the soluble fraction of AgNPs, allowing to estimate the role of cell-NP contact on intracellular Ag ions (Figure 4).

**Figure 5:**
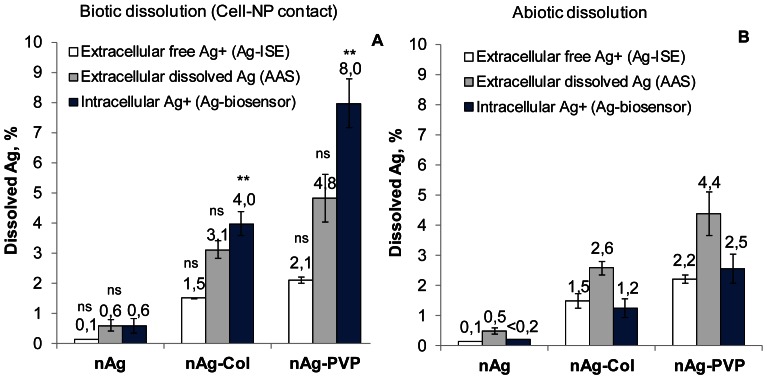
Dissolved, free and intracellular concentrations of Ag in biotic and abiotic conditions. A: Dissolved, free and intracellular Ag after 4-h direct cell-NP contact. Intracellular Ag^+^ was quantified from bioluminescent response of *E. coli* MC1061 (pSLcueR/pDNPcopAlux) to AgNPs suspension. Extracellular dissolved Ag was measured by atomic absorption spectroscopy (AAS) and free Ag^+^ was measured by Ag-ion-selective electrode (Ag-ISE) from the test medium after settling of NPs and bacterial cells by ultracentrifugation at 390 000 g for 1 hour. B: Dissolved, free and intracellular Ag in abiotic conditions. Extracellular dissolved Ag (AAS), extracellular free Ag^+^ (Ag-ISE) and intracellular Ag^+^ (Ag-biosensor *E. coli* MC1061 (pSLcueR/pDNPcopAlux)) were measured from the test medium after settling of NPs by ultracentrifugation at 390 000 g for 1 hour. Data represent the average ± SD (n = 3). **p<0.01, ns – not significant (p>0.05) compared to abiotic dissolution according to two-tailed unpaired *t*-test.

The results from AAS revealed that all AgNPs dissolved slightly more in the presence of bacteria than in abiotic conditions, but this effect was not statistically significant (n = 3, p>0.05) (Figure 5, grey bars). The results obtained using Ag-ISE showed also that compared to abiotic conditions no additional free Ag ions appeared in the test medium when AgNPs were incubated with bacterial cells (Figure 5 A and B, white bars). In both, biotic and abiotic conditions, the extracellular fraction of dissolved Ag determined with AAS exceeded the fraction determined with Ag-ISE about twice. This difference was expected, because Ag-ISE determines only free Ag ions but AAS determines also the complexes of Ag ions with the low-molecular-weight (<5 kDa) components of the test medium that were too small to settle during ultracentrifugation.

While no additional extracellular dissolution of AgNPs was detected in biotic exposure conditions, there was a significantly increased uptake of Ag ions *via* cell-NP interaction. Specifically, in case of direct contact between AgNPs and *E. coli* MC1061 (pSLcueR/pDNPcopAlux) (i.e., biotic conditions) the biosensor cells internalized about 3 times more Ag ions than were internalized when the biosensor was exposed to ultracentrifuged supernatants of AgNPs (Figure 5A *versus* 5B, blue bars). This result reveals the importance of direct contact of AgNPs with bacterial cells and demonstrates that the extracellular concentration of Ag ions in the test medium may underestimate the effective intracellular concentrations and hence, antibacterial potency of Ag ions from AgNPs.

**Figure 6:**
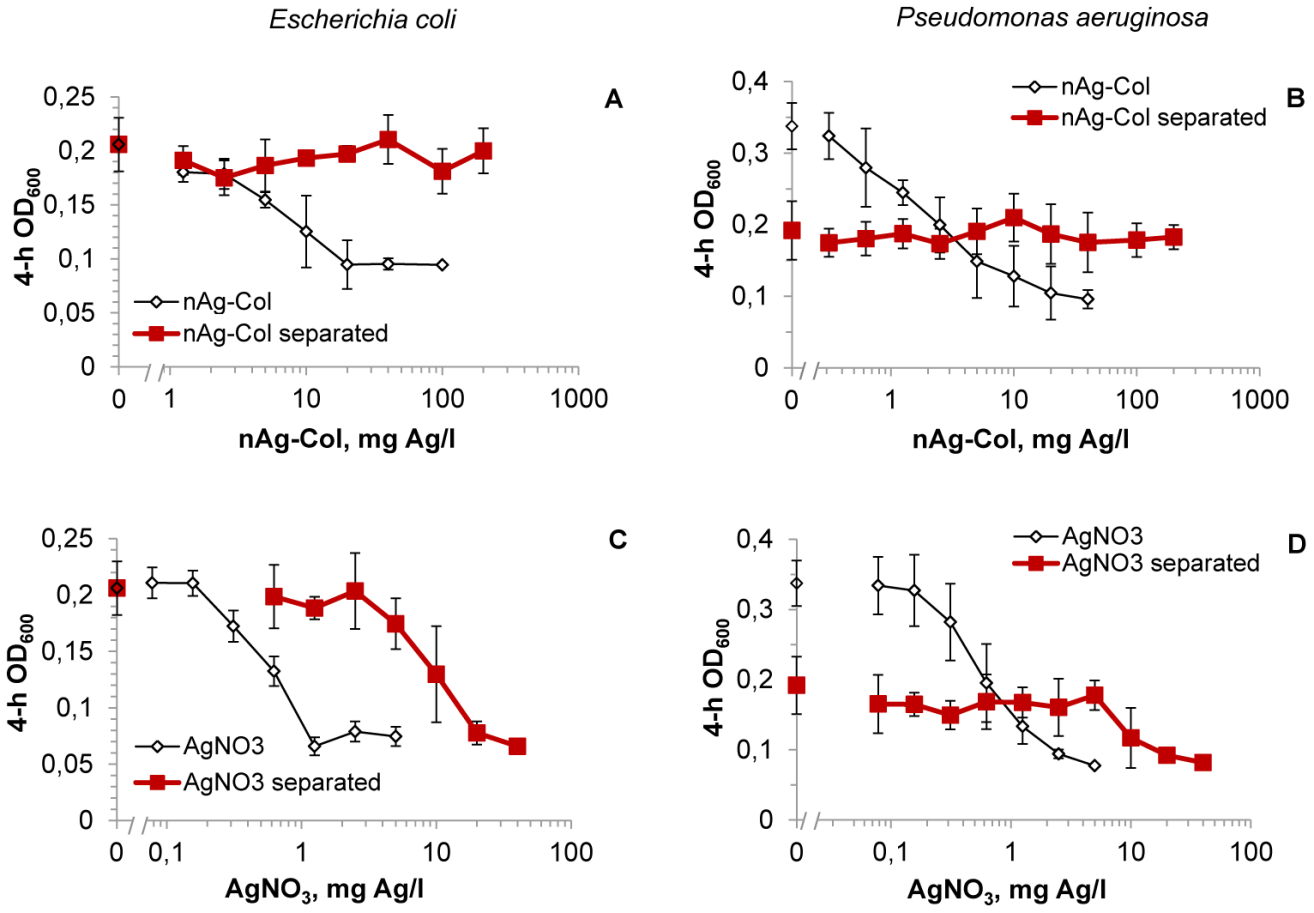
Bacterial growth after 4-h exposure to collargol directly and through the dialysis membrane. *Escherichia coli* MC1061 (A, C) and *Pseudomonas aeruginosa* DS10-129 (B, D) upon exposure to collargol (nAg-Col; A, B) after 4 h in half-strength NaCl-free LB at 30°C in the direct contact with nanoparticles (rectangle) or being separated from particles by 20 kDa (4 nm) cut-off membrane (diamond). AgNO_3_ was handled analogously to collargol and was used as a control (C, D). Data represent the average ± SD (n = 4).

To further demonstrate that the intimate surface contact of AgNPs with bacterial cells increases the internalization of particle-associated Ag ions and, therefore, the effective toxicity of AgNPs, comparative growth inhibition assays were carried out with *E. coli* and *P. aeruginosa* cells either directly exposed to nAg-Col particles or separated from the nanoparticles by a particle-proof dialysis membrane (cut-off 20 kDa∼4 nm; Figure S6). When *E. coli* cells were in direct contact with AgNPs, the 4-h EC_50_ value for nAg-Col was around 10 mg/l (Figure 6A). However, when nAg-Col was separated by a membrane, the toxic effect was not observed even at 200 mg/l (Figure 6A, red line) i.e, there was a >20-fold reduction in toxicity. Similarly, membrane-separated nAg-Col did not exhibit any toxic effects to *P. aeruginosa* (Figure 6B). Interestingly, the optical density of *P. aeruginosa* culture was lower in the presence of the dialysis membrane: when the OD_600_ values without the membrane were around 0.35 then in the presence of the membrane the maximum OD_600_ was only 0.2 (Figure 6B). This was most probably because *P. aeruginosa* cells tended to attach to the dialysis membrane immediately after the contact (data not shown).

In the experimental setup used to separate nAg-Col by the dialysis membrane, we did not establish equilibrium for Ag ions between the membrane-separated phases beforehand, because it would have caused additional dissolution of nAg-Col complicating the interpretation of the results. Therefore, most likely there was an unequal distribution of Ag ions between the two membrane-separated phases. To evaluate the efficiency of Ag ions' diffusion through the membrane during the 4-hour test, bacteria were also exposed to AgNO_3_ either directly or through a dialysis membrane. Without the dialysis membrane, the complete inhibition of *E. coli* growth after 4 hours of incubation was observed at 1.25 mg Ag/l of AgNO_3_. However, when the bacteria were exposed to AgNO_3_ through a membrane, 20 mg Ag/l of AgNO_3_ was required to inhibit bacterial growth (Figure 6C, D). Thus, the dialysis membrane caused 16-fold reduction in toxicity of AgNO_3_. Taking this into account we estimated that if the toxicity of nAg-Col would have been mediated only by the extracellularly dissolved Ag ions, the membrane-separated nAg-Col should have inhibited *P. aeruginosa* and *E. coli* growth starting from 80 or 160 mg/l, respectively. However, this was not the case (Figures 6A, B), additionally confirming that in the absence of direct contact with Ag-particles the effective intracellular concentration of Ag ions in bacterial cells was lower.

### Pseudomonas aeruginosa Cells Co-precipitate with Collargol

**Figure 7:**
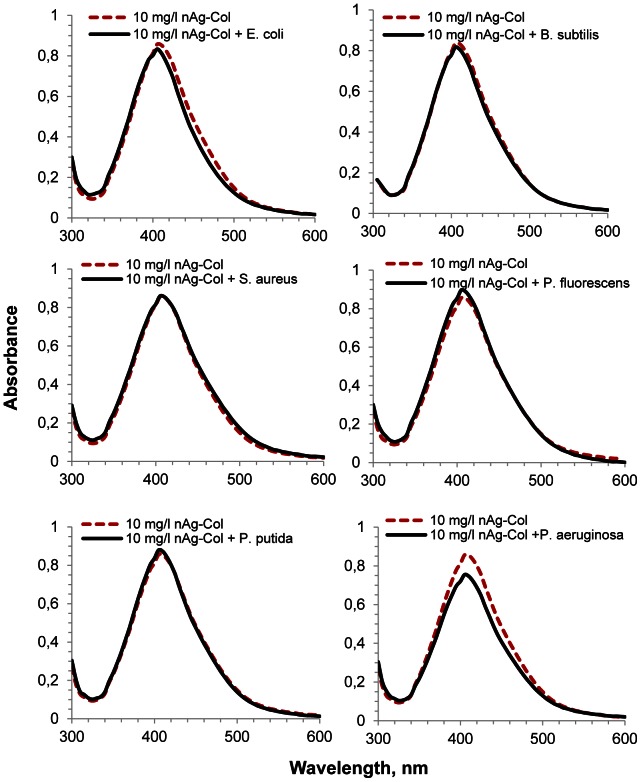
UV-visible absorption spectra of nAg-Col suspensions (10 mg Ag/l) with or without bacteria. UV-Vis spectra of AgNPs in half-strength NaCl-free LB medium without bacterial cells (dashed red line) or after exposure of bacterial cells to nAg-Col (solid line). Before measurement, bacterial cells and cell-associated nanoparticles were removed by centrifugation at 4 000 g for 5 minutes. The difference between dashed and continuous lines shows the co-precipitation of nAg-Col particles with bacterial cells. The representative figures from the three independent experiments are shown.

Since the toxicity of AgNPs was apparently mediated by the cell-NP contact, which depends on specific surface properties of each bacterium, the potential of different bacterial strains to attach to the surface of AgNPs was studied. For that, all the six test bacteria were mixed with nAg-Col, immediately settled by centrifugation and UV-Vis spectra of the obtained supernatants were measured. The plasmon absorption band near 400 nm that is proportional to the concentration of metallic nanosized Ag [25] enabled to estimate the fraction of nAg-Col which remained unadsorbed to bacterial cells. Before the experiment we verified that the addition of bacterial culture to nAg-Col suspension had no effect on the specific plasmon resonance peak of the nanoparticles (Figure S7). While no co-precipitation of AgNPs with *E. coli*, *B. subtilis*, *S. aureus*, *P. fluorescens* and *P. putida* cells was observed (the UV-Vis absorption spectra before and after centrifugation were similar), there was a significant co-precipitation of nAg-Col and *P. aeruginosa* cells (Figure 7). According to the peak of UV-Vis spectrum, 9.2±2.6% of nAg-Col was readily sorbed to the cell surface and co-precipitated with *P. aeruginosa* cells during the centrifugation. This observation suggests that *P. aeruginosa* cells had higher affinity to AgNPs than the other tested bacterial strains. We assume that the adhesion of *P. aeruginosa* cells to nAg-Col particles was responsible for the high antibacterial potency of nAg-Col particles towards this pathogenic bacterium (Figure 2).

## Discussion

It is generally acknowledged that the toxicity of AgNPs depends on the release of Ag^+^ ions, which interact with -SH groups of membrane-bound enzymes and proteins, uncoupling the respiratory chain of bacteria [26], [27] and disrupting bacterial cell membranes [28]. This hypothesis implies that the toxic effects of nanosilver are proportional to the activity of free Ag ions released from AgNPs into extracellular solution, assuming the equal distribution of Ag^+^ on the surface of nanoparticles, in the solution and inside bacterial cell. This explanation of the toxicity does not consider the impact of direct interaction of AgNPs with bacterial cells.

In this study we characterized three differently coated AgNPs and quantified their effects on the growth and viability of six bacterial strains. To measure Ag dissolved from AgNPs, we used three different techniques that enabled to distinguish between the fractions of extracellular dissolved Ag, extracellular free Ag ions and intracellular Ag ions.

The sensitivity of all the six used bacterial strains towards AgNO_3_ was relatively similar. The difference between the most and the least susceptible bacterial strain (*P. putida* and *S. aureus*, respectively) was only 4-fold. At the same time there was 130-fold difference between the toxicity of protein-coated nAg-Col to different bacterial strains (*P. aeruginosa vs. S. aureus*, Figure 2). It was evident that Ag dissolved from AgNPs played a strong antibacterial role as the dissolution rate of AgNPs (Table 1) correlated well with their toxicity to bacteria (Figure 2). However, the **extracellular** dissolution alone did not explain the orders of magnitude difference in toxicity of AgNPs to different bacterial strains. At the same time, the toxicity of AgNPs to *E. coli* MC1061 was fully predicted by **intracellular** effective fraction of Ag ions (Figure 3) determined by Ag-biosensor *E. coli* MC1061 (pSLcueR/pDNPcopAlux). Further, the results of Ag-biosensor revealed that 3–4 times more Ag ions were internalized by *E. coli* cells upon direct contact with nanoparticles compared to the particle-free supernatants of respective ultracentrifuged suspensions (Figure 5). These results showed that intracellular Ag ion is the single and ultimate cause of the antibacterial action of studied AgNPs and that the concentrations of the former depended mainly on two factors: extracellular dissolution (Table 1) and cell-NP contact (Figures 5–7).

There are plenty of data showing that AgNPs are more toxic to bacterial cells than dissolved silver released from these NPs [16], [29], [30]. In the literature, additional particle-attributed toxicity is usually explained by the production of reactive oxygen species (ROS) by the particle surface and/or mechanical damage of bacterial cell membranes [10], [31]. At the same time it has been clearly shown that AgNPs that lacked oxidized surfaces (and therefore were not dissolving) proved also not toxic to bacteria [12], [13], excluding the involvement of ROS and cell membrane damage by particles *per se*. These seemingly contradicting results can be explained by additional dissolution of AgNPs in the close vicinity of bacterial cell envelope upon cell-NP interaction. The importance of cell-NP contact has been also suggested and discussed previously [32], [33], [34]. Using *E. coli*, McQuillan et al. [18] noticed that Ag^+^ from AgNPs induced 2–3-fold up-regulation of Ag^+^-response genes *copA*, *cueO* and *cusA* compared to AgNO_3_ and referred to this effect as “nanoparticle-enhanced silver ion stress”. Recently, Taglietti et al. [35] suggested that overall bactericidal effect of glutathione-coated nAg to *E. coli* and *S. aureus* depended on two factors: the release of Ag ions from the NPs referred to by authors as “long-distance mechanism” and the nanomechanical damage of bacterial membranes (“short-distance mechanism”). Hereby, we complement these studies and show that both, “long-distance” and “short-distance” effects of AgNPs are in fact the action of Ag ions. It should be pointed out that the release and adverse effects of “long-distance Ag ions” (extracellular dissolution) are well-known and can be easily quantified using AAS or ICP. In contrast, the contribution of “short-distance Ag ions” (dissolution at cell-NP interface) to the toxicity is a complex process that is often overlooked and is rather reported in terms of secondary effects such as damage to bacterial cell wall.

The enhanced toxicity of particle-associated Ag^+^ through the cell-NP interaction assumingly involves the release of silver ions in the close proximity of bacterial outer surface and also possible cellular internalization of AgNPs. In the aqueous environment, the surface of most nanoparticles is electrically charged and therefore, may attract counter-ions [32], [36], [37]. Most of the nanoparticles as well as AgNPs used in our study encounter negative charge (measured as ζ-potential, Table 1) in aqueous media [37]. Thus, they have the potency to attract counter-ions e.g., Ag^+^ and H^+^ cations into their diffuse layer, e.g., by chemisorption [38]. Upon direct contact with negatively charged bacterial cells (Table S3), the increased concentration of both, Ag^+^ and H^+^ ions on the particle surface would cause additional damage to bacterial cell membranes, because of the higher local concentration of Ag ions and higher local dissolution rate of AgNPs upon low pH. It is well known that Ag ions destabilize the membranes of both Gram–positive and Gram–negative bacteria [14], [28], [39]. Such local intensive influx and damaging action of Ag ions from AgNPs may create pits in bacterial cells walls, disrupting cellular integrity, facilitating the internalization of AgNPs [18], [31], [40] and causing a myriad of secondary effects such as Ag^+^-triggered accumulation of intracellular ROS and damage of vital biomolecules. High toxicity of all forms of nanoparticulate silver (Figure 2), especially collargol, to *P. aeruginosa* supports this hypothesis, as *P. aeruginosa* adhered to nAg-Col stronger than the rest of the tested bacterial species (Figure 7). The attachment of microbes to a surface is also an initial phase of the biofilm formation [41]. *P. aeruginosa* strains tend to form biofilms in immunocompromised hosts and in human implants, exhibiting increased tolerance to antibiotics in biofilm state [42]. On the basis of our results we suggest that nanoparticulate silver may be especially efficient in inhibiting initial cell attachment and thus, retarding the formation of bacterial biofilms as was already shown empirically for *Pseudomonas aeruginosa* PAO1 [43].

All the above indicate that the net antibacterial effect of the studied nanosilver depends exclusively on the effective concentration of silver ions inside the bacterial cells, which is determined by two main factors (i) extracellular dissolution of AgNPs and (ii) dissolution of AgNPs on particle-cell interface. Notably, as only a limited number of spherical AgNPs were utilized in this study, further research may be needed to understand whether the cell-NP interaction is also responsible for the shape-dependent [15], surface charge dependent [44] or surface-defect and crystallinity-dependent [45] toxicity of AgNPs.

Taken together, this paper is one of the first to provide quantitative evidence on the role of cell-NP contact in the toxicity of AgNPs and to demonstrate that enhanced “particle-specific” toxicity debated in the literature is manifested *via* increased intracellular effective concentration of Ag ions – the ultimate cause of toxicity of AgNPs. The exact physico-chemical and cellular mechanisms underlying this phenomenon remain, however, still hypothetical and need further research.

## Supporting Information

Figure S1 **Number and share of articles on silver nanoparticles for six bacterial species in ISI WoS.** Keywords used for the search and respective number of retrieved papers were as follows: ‘silver nanoparticles’ and ‘Escherichia coli’ (1162 papers); ‘silver nanoparticles’ and ‘Staphylococcus aureus’ (554 papers), ‘silver nanoparticles’ and ‘Pseudomonas aeruginosa’ (184 papers); ‘silver nanoparticles’ and ‘Bacillus subtilis’ (114 papers); ‘silver nanoparticles’ and ‘Pseudomonas putida’ (11 papers); ‘silver nanoparticles’ and ‘Pseudomonas fluorescens’ (10 papers). Total: 2035 papers. Search made: 06.12.2012.
(DOCX)

Figure S2 **Primary size distribution of silver nanoparticles.** A: PVP-coated nAg-PVP, B: protein-coated nAg-Col and C: uncoated nAg. Sizes of the silver nanoparticles were measured from TEM micrographs (Figure 1); altogether 65 particles were measured.
(DOCX)

Figure S3
**Response of Ag-biosensor to silver formulations.** Representative calibration curves of Ag-biosensor *Escherichia coli* MC1061 (pSLcueR/pDNPcopAlux) for AgNO_3_ (A–C), nAg-PVP (A), nAg-Col (B) and nAg (C) are shown. The lines represent the calibration plots for the linear regression between log(C)−log(indBL), whereas C is concentration of Ag (mg/l) in the samples and indBL is a fold induction of bioluminescence in Ag-biosensor in response to intracellular Ag. Linear range of the regression and regression equations are shown. Intracellular Ag was determined by using the linear regression equation for AgNO_3_, which was considered 100% bioavailable and was used as a standard. Calculations were performed at BL = 10. An example for quantification of intracellular dissolution of nAg-PVP is given below:
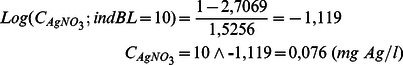


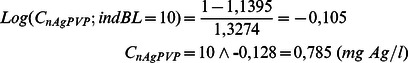


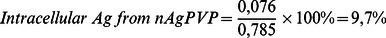

(DOCX)

Figure S4 **Ultraviolet - visible (UV-Vis) wavelength absorption spectra for silver nanoparticles.** UV-Vis spectra for 10 mg/l nAg-Col suspension (A) and 10 mg/l nAg-PVP suspension (B) immediately after dispersion in the test medium (half-strength NaCl-free LB) (solid line) and after 4-h incubation (dashed green line) are shown. The UV-Vis measurement could not be conducted for the uncoated nAg NPs due to their quick sedimentation. The plasmon absorption peak at 405 nm is proportional to the concentration of metallic nanosized Ag and the decrease of this peak indicates dissolution of AgNPs. The absence of shift of plasmon absorption peak indicates that AgNPs do not aggregate. Insets in the Figure S4 indicate dissolution of AgNPs measured by atomic absorption spectroscopy (AAS) from the ultracentrifuged (390 000 g for 60 minutes) supernatants of AgNPs. During 4-h incubation, AgNPs dissolved but released Ag ions were complexed by the components of test medium that settled during ultracentrifugation.
(DOCX)

Figure S5 **Growth inhibition of bacterial strains by silver nanoparticles.** Growth inhibition of *Escherichia coli* (A), *Bacillus subtilis* (B), *Staphylococcus aureus* (C), *Pseudomonas fluorescens* (D), *Pseudomonas putida* (E) and *Pseudomonas aeruginosa* (F) by uncoated Ag nanoparticles (nAg; triangle), collargol (nAg-Col; rectangle), PVP-coated Ag nanoparticles (nAg-PVP; cross) or AgNO_3_ (diamond) after 4-h incubation in half-strength NaCl-free LB medium at 30°C. The representative figures from three biological replicates are shown.
(DOCX)

Figure S6 **Setup of the dialysis membrane test.** Bacterial cells were separated from AgNPs or AgNO_3_ by 20 kDa (about 4 nm) dialysis membrane (Slide-A-Lyzer MINI Dialysis Device, 20K MWCO, Thermo Scientific). 400 µl of bacterial suspension was pipetted into the wells, polypropylene cups with the dialysis membrane on the bottom were inserted into the wells and 400 µl of AgNPs, AgNO_3_ or DI water (control) was pipetted into the cups. During the optical density measurements the cups were removed.
(DOCX)

Figure S7 **UV-visible absorption spectra of nAg-Col suspensions (10 mg Ag/l) with or without bacteria.** UV-Vis spectra of AgNPs in half-strength NaCl-free LB medium without bacterial cells (dashed red line) or after exposure of nAg-Col to bacterial cells (solid line). No separation of particles and/or bacterial cells was done before the measurement. The experiment is a control for Figure 7 to exclude the interference of bacterial cells with the measurements of UV-Vis absorption spectra of nAg-Col. Addition of bacterial culture had no significant effect on the UV-Vis spectra of nAg-Col.
(DOCX)

Table S1 **Characteristics of the bacterial strains used in this study.**
(DOCX)

Table S2 **Minimum bactericidal concentration (MBC mg Ag/l) of silver nanoparticles and AgNO_3_ to six bacterial strains.** nAg states for uncoated AgNPs, nAg-PVP for PVP-coated AgNPs and nAg-Col for collargol. Bacteria were incubated with different concentrations of Ag-compounds in half-strength NaCl-free LB medium at 30°C for 4 h. Then, 3 µl of the test sample was pipetted onto agarized LB plates, incubated at 30°C for 24 h and visually inspected for the growth. The lowest tested concentration that completely inhibited the visible growth of bacteria was designated as a MBC.
(DOCX)

Table S3 **ζ-potential of bacterial cells in half-strength NaCl-free LB medium.**
(DOCX)
