# Supplementary figures and images for: Particle-Cell Contact Enhances Antibacterial Activity of Silver Nanoparticles
Source: PLoS One. 2013 May 30;8(5):e64060. doi: 10.1371/journal.pone.0064060 (PMC3667828; doi:10.1371/journal.pone.0064060)

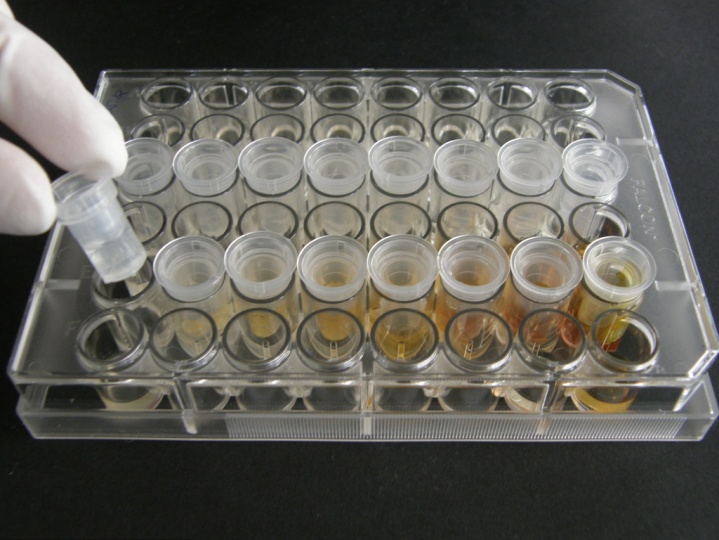


**Figure S6**

Supplement: Figure S6 — Setup of the dialysis membrane test. Bacterial cells were separated from AgNPs or AgNO3 by 20 kDa (about 4 nm) dialysis membrane (Slide-A-Lyzer MINI Dialysis Device, 20K MWCO, Thermo Scientific). 400 µl of bacterial suspension was pipetted into the wells, polypropylene cups with the dialysis membrane on the bottom were inserted into the wells and 400 µl of AgNPs, AgNO3 or DI water (control) was pipetted into the cups. During the optical density measurements the cups were removed. (DOCX) [file pone.0064060.s006.docx]
