# Supplementary material for: Particle-Cell Contact Enhances Antibacterial Activity of Silver Nanoparticles
Source: PLoS One. 2013 May 30;8(5):e64060. doi: 10.1371/journal.pone.0064060 (PMC3667828; doi:10.1371/journal.pone.0064060)
Supplement: Table S1 — Characteristics of the bacterial strains used in this study. (DOCX) [file pone.0064060.s008.docx]

**Table S1**

| **Bacterial strain** | **Genotype or description** | **Reference** |
| --- | --- | --- |
| *Escherichia coli* MC1061 | *araD*139 Δ*(ara, leu)*7697 Δ*lacX*74 *galU* *galK* *hsdR*2 *strA* *mcrA* *mcrB*1 | ^1^ Casadaban and Cohen, 1980 |
| *Bacillus subtilis* BR151 | *trpC*2 *lys*-3 *metB*10 | ^2^ Young et al., 1969 |
| *Staphylococcus aureus* RN4220 | *rsbU*- *agr*- | ^3^ Kreiswirth et al., 1983 |
| *Pseudomonas fluorescens* OS8 | Rifampicin^r^, isolated from toluates-contaminated soil | ^4^ Sarand et al., 2000 |
| *Pseudomonas putida* KT2440 | *rmo- mod+* | ^5^ Bagdasarian et al., 1981 |
| *Pseudomonas aeruginosa*  DS10-129 | Ampicillin^r^, kanamycin^r^, isolated from diesel-contaminated site | ^6^ Rahman et al., 2000 |

^1^ Casadaban MJ, Cohen SN (1980) Analysis of gene control signals by DNA fusion and cloning in *Escherichia coli*. J Mol Biol 138: 179–207.

^2^ Young FE, Smith C, Reilly BE (1969) Chromosomal location of genes regulating resistance to bacteriophage in *Bacillus subtilis*. J Bacteriol 98: 1087–1097.

^3^ Kreiswirth BN, Lofdahl S, Betley MJ, O'Reilly M, Schlievert PM et al. (1983) The toxic shock syndrome exotoxin structural gene is not detectably transmitted by a prophage. Nature. 305: 709–712.

^4^ Sarand I, Haario H, Jorgenson KS, Romantschuk M (2000) Effect of inoculation of a TOL plasmid containing mycorrhizosphere bacterium on development of Scots pine seedlings, their mycorrhizosphere and the microbial flora in m-toluate-amended soil. FEMS Microbiol Ecol 31: 127–141.

^5^ Bagdasarian M, Lurz R, Ruckert B, Franklin FC, Bagdasarian MM et al. (1981) Specific-purpose plasmid cloning vectors. II. Broad host range, high copy number, RSF1010-derived vectors, and a host-vector system for gene cloning in *Pseudomonas*. Gene 16: 237–247.

^6^ Rahman KSM, Rahman TJ, Lakshmanaperumalsamy P, Marchant R, Banat IM (2002) Emulsification potential of bacterial isolates with a range of hydrocarbon substrates. Acta Biotechnol 23: 335–345.
