# Supplementary material for: Particle-Cell Contact Enhances Antibacterial Activity of Silver Nanoparticles
Source: PLoS One. 2013 May 30;8(5):e64060. doi: 10.1371/journal.pone.0064060 (PMC3667828; doi:10.1371/journal.pone.0064060)
Supplement: Table S2 — Minimum bactericidal concentration (MBC mg Ag/l) of silver nanoparticles and AgNO3 to six bacterial strains. nAg states for uncoated AgNPs, nAg-PVP for PVP-coated AgNPs and nAg-Col for collargol. Bacteria were incubated with different concentrations of Ag-compounds in half-strength NaCl-free LB medium at 30°C for 4 h. Then, 3 µl of the test sample was pipetted onto agarized LB plates, incubated at 30°C for 24 h and visually inspected for the growth. The lowest tested concentration that completely inhibited the visible growth of bacteria was designated as a MBC. (DOCX) [file pone.0064060.s009.docx]

| **Bacterial strain** | **Gram** | **4-h minimum bactericidal concentration**  **(MBC), mg Ag/l** | | | |
| --- | --- | --- | --- | --- | --- |
|  |  | **nAg** | **nAg-Col** | **nAg-PVP** | **AgNO_3_** |
| *Escherichia coli* MC1061 | **G−** | >100 | 40 | 40 | 5 |
| *Bacillus subtilis* BR151 | **G+** | >100 | 40 | 20 | 5 |
| *Staphylococcus aureus* RN2440 | **G+** | >100 | 100 | >100 | 40 |
| *Pseudomonas fluorescens* OS8 | **G−** | >100 | 100 | 40 | 5 |
| *Pseudomonas putida* KT2440 | **G−** | >100 | 40 | 40 | 5 |
| *Pseudomonas aeruginosa* DS10-129 | **G−** | 100 | 5 | 20 | 5 |

**Table S2**
