# Supplementary material for: Particle-Cell Contact Enhances Antibacterial Activity of Silver Nanoparticles
Source: PLoS One. 2013 May 30;8(5):e64060. doi: 10.1371/journal.pone.0064060 (PMC3667828; doi:10.1371/journal.pone.0064060)
Supplement: Table S3 — ζ-potential of bacterial cells in half-strength NaCl-free LB medium. (DOCX) [file pone.0064060.s010.docx]

**Table S3.**

| **Bacterial strain** | **ζ-potential** |
| --- | --- |
| *Escherichia coli* MC1061 | -32,9 |
| *Bacillus subtilis* BR151 | -35,2 |
| *Staphylococcus aureus* RN2440 | -14,0 |
| *Pseudomonas fluorescens* OS8 | -8,4 |
| *Pseudomonas putida* KT2440 | -9,1 |
| *Pseudomonas aeruginosa* DS10-129 | -23,9 |
